# A Pilot Study of VGGish-CNN as a Model for the Classification of Parkinson’s Disease from a Control Group Using Speech Impairment, Integrated with Explainable Artificial Intelligence

**DOI:** 10.3390/bioengineering13091058

**Published:** 2026-09-11

**Authors:** Mehdi Rashidi, Syed Adil Hussain Shah, Marco Greco, Marta Lorenzo, Andrea Buccoliero, Serena Arima, Angela Lupo, Filomena My, Chiara Coppola, Marcello Donzella, Alberto Argentiero, Michele Maffia

**Affiliations:** 1 Department of Mathematics and Physics ”E. De Giorgi”, University of Salento, Via Lecce-Arnesano, 73100 Lecce, Italy; mehdi.rashidi@unisalento.it; 2 PolitoBioMed Lab, Department of Mechanical and Aerospace Engineering, Politecnico di Torino, 10129 Torino, Italy; syedadilhussain@gpi.it; 3 Department of Research and Development (R&D), GPI SpA, 38123 Trento, Italy; andrea.buccoliero@gpi.it (A.B.);; 4 Department of Experimental Medicine, University of Salento, Via Lecce-Monteroni, 73100 Lecce, Italychiara.coppola@unisalento.it (C.C.); 5 Division of Neurology, Vito Fazzi Hospital, 73100 Lecce, Italy; martatnfp@gmail.com (M.L.); angela87lupo@gmail.com (A.L.); filomenamy28@gmail.com (F.M.); 6 Human Science Department, University à Degli Studi di Verona, Lungadige Porta Vittoria, 17, 37129 Verona, Italy; 7 Department of Human and Social Sciences, University of Salento, 73100 Lecce, Italy; serena.arima@unisalento.it; 8 Department of Medicine and Surgery, University of Parma, 43121 Parma, Italy

**Keywords:** Parkinson’s disease, convolutional neural network, explainable artificial intelligence, voice impairment

## Abstract

**Introduction:** Voice-based digital biomarkers have emerged as a promising, non-invasive approach for the early detection and monitoring of neurodegenerative disorders, particularly Parkinson’s disease (PD). Although voice recordings can be acquired easily using mobile health technologies, their integration into routine clinical practice remains limited due to challenges related to model interpretability and clinical validation. This study aimed to investigate the diagnostic potential of voice recordings acquired through the TALIA smartphone and web-based platform and to improve model transparency using explainable artificial intelligence (XAI) techniques. **Methods:** Voice recordings from participants with PD and control group (CG) were acquired over a six-month period using the TALIA digital-health platform at Vito Fazzi Hospital in Lecce, Italy. The data were evaluated cross-sectionally at the recording level. Sustained vowel (/a/) phonations were preprocessed and transformed into spectrogram images. A transfer-learning framework based on the pre-trained VGGish convolutional neural network (VGGish-CNN) was developed to classify PD and CG voice recordings. To enhance interpretability, explainable artificial intelligence (XAI) methods, including Local Interpretable Model-Agnostic Explanations (LIME) and Occlusion Sensitivity, were integrated to identify the spectro-temporal regions contributing most strongly to model predictions. **Results:** The proposed VGGish-CNN framework demonstrated excellent classification performance in distinguishing PD from CG. The model achieved an accuracy of 0.93, precision of 0.91, recall of 0.95, F1-score of 0.93, loss of 0.16, and an area under the receiver operating characteristic curve (AUC) of 0.987. XAI analyses provided qualitative, sample-specific visualizations of the spectro-temporal regions contributing to individual model predictions, thereby improving the transparency and interpretability of the deep-learning model. **Conclusions:** The findings demonstrate that a transfer-learning approach based on VGGish-CNN could achieve promising recording-level classification performance in distinguishing voice recordings from participants with PD and CG within this pilot dataset. Furthermore, XAI techniques provide qualitative insight into the model’s decision-making process. These preliminary findings support further investigation of voice-based approaches for PD screening in larger cohorts using participant-level and external validation.

## 1. Introduction

Voice biomarkers, which are derived from voice using artificial intelligence (AI), can be nominated as promising way for distinguishing between PD and CG [1]. Speech alterations may manifest during the prodromal phase of PD, potentially aiding neurologists in early detection of PD [1,2]. Voice collection is non-invasive, relatively inexpensive, and suitable for remote monitoring, making it attractive for digital-health applications [3]. PD is a neurodegenerative disorder associated with intracellular α-synuclein accumulation and substantial progressive neuronal loss in the substantia nigra pars compacta (SNc) [4]. Currently, clinical detection is time-consuming, and untrained eyes can often only detect PD after several years, causing a loss of the golden time for intervention. Thus, digital-health approaches, such as AI-driven speech analysis, can help detect PD earlier, possibly even prior to the significant diminishing of neurons in the SNc [5]. According to research, up to 70% of people with PD may have speech impairment, which can lead to social interaction difficulties and depression in these people [6]. Speech and language alterations have been observed prior to the manifestation of motor signs among PD patients, at least ten years before [7]. Consequently, increasing attention has been directed toward AI-based analysis of speech and voice as potential digital biomarkers for PD [8]. Carron et al. [9] analyzed sustained vowel /a/ in uncontrolled and unsupervised situations in a home-based database by recruiting 30 PD and 30 CG. Although six different classifiers were evaluated, the Passive Aggressive classifier demonstrated a marked difference in performance based on the data source. It achieved high accuracy (area under the receiver operating characteristic curve or AUC between 0.8 and 0.9) using speech recordings taken in a controlled setting, but its accuracy was significantly lower (AUC between 0.6 and 0.7) when tested with the smartphone-recorded (mPower) database [10]. In recent years, many papers have been published that developed algorithms to detect PD from CG in the early detection of the disease. However, several methodological challenges remain. Firstly, they used internal cross-validation without clearly defining the train and test parts [11], whereas clearly splitting voice files into training, test, and validation sets can help avoid overfitting. Secondly, in many papers, they relied on traditional imaging methods to detect PD or clinical studies, while nowadays, researchers utilized CNN models for Parkinson’s detection from CG that rely not only on CNN models and transfer learning but also on XAI to enhance clinical interpretability [12]. Little et al. were among the pioneering researchers to demonstrate that dysphonia-related acoustic measures could be used with support vector machine classifiers to distinguish PD from control participants [13]. Iyer et al. [11] studied voice analysis by CNN-Inception V3 as transfer learning which was pre-trained on the ImageNet [14] database because it has been shown to adapt successfully to medical imaging problems through transfer learning with high accuracy [15]. Although this study demonstrated the potential of transfer learning for voice-based classification, explainability methods were not incorporated to investigate the spectro-temporal regions contributing to individual predictions. The Unified Parkinson’s Disease Rating Scale (UPDRS) is widely used to characterize the clinical severity of PD and includes assessment of speech-related impairment [16,17].

Shen et al. also utilized Iyer’s raw voice data [18] to optimize results and interpret them using XAI, but they did not use transfer learning such as InceptionV3 as pre-trained data, as done in previous research, to obtain real-world achievement. Moreover, their AUC results achieved scores between 0.41 and 0.91 among combined models [12]. Alissa et al. reported that a convolutional neural network (CNN) was used to extract and analyze voice features, achieving a diagnostic accuracy of 0.935 [19], which demonstrates a broader shift in the field. These studies collectively underline the importance of transitioning from traditional machine learning to more advanced deep-learning models, such as CNNs, to significantly enhance diagnostic accuracy [20]. Data scientists have been using Boosted Decision Trees or extreme Gradient Boosting (XGBoost) in discriminating Parkinson’s from participants without PD, achieving promising outcomes, including 0.90–0.95 of accuracy [21]. These gradient-boosted models outperformed the Random Forest and logistic regression models evaluated in their study [22]. While deep-learning (DL) models have shown promise in classifying and detecting acoustic signals, the limited interpretability of their decision-making mechanisms creates difficulties in identifying the specific features that influence model predictions [23]. Explainable artificial intelligence (XAI) techniques are increasingly used to determine which input features or regions contribute to model predictions, thereby improving model interpretability [12,24]. Different XAI approaches can be applied for this purpose, such as SHapley Additive Explanations (SHAP), Local Interpretable Model-Agnostic Explanations (LIME), and Occlusion Sensitivity. For spectrogram-based classification, LIME and Occlusion Sensitivity can visualize sample-specific spectro-temporal areas that influence individual model outputs. Nevertheless, these highlighted regions should not be regarded as clinically meaningful biomarkers unless they are systematically confirmed in larger datasets [24].

Moreover, the performance of the proposed VGGish-CNN framework was compared to acoustic-feature-based machine-learning approaches tested on the same clinical cohort and voice dataset. The comparison was made to investigate whether automatic representation learning with VGGish can achieve competitive performance compared to traditional methods involving explicit acoustic feature extraction and feature selection. Because the two pipelines were built on different data-partitioning schemes, this comparison is considered more descriptive than a controlled head-to-head evaluation.

In this pilot study, voice samples from participants with PD and CG were obtained through the TALIA smartphone and web platforms during a six-month collection period. The resulting recordings were evaluated using a cross-sectional, recording-level design. A transfer-learning framework based on the pre-trained VGGish-CNN was combined with LIME and Occlusion Sensitivity to classify sustained /a/ recordings and qualitatively interpret individual predictions. The main contributions of this work are therefore: (1) the analysis of an original clinical voice dataset collected through the TALIA digital-health platform, (2) the application of VGGish-based transfer learning for PD/CG voice classification, and (3) the integration of LIME and Occlusion Sensitivity to improve the transparency of the resulting predictions. The study is intended as a pilot recording-level evaluation, and its findings should not be interpreted as evidence of participant-level generalizability [25]; this paper also uses XAI techniques to further enhance both the prediction and interpretation of the CNN models.

## 2. Materials and Methods

**Figure 1:**
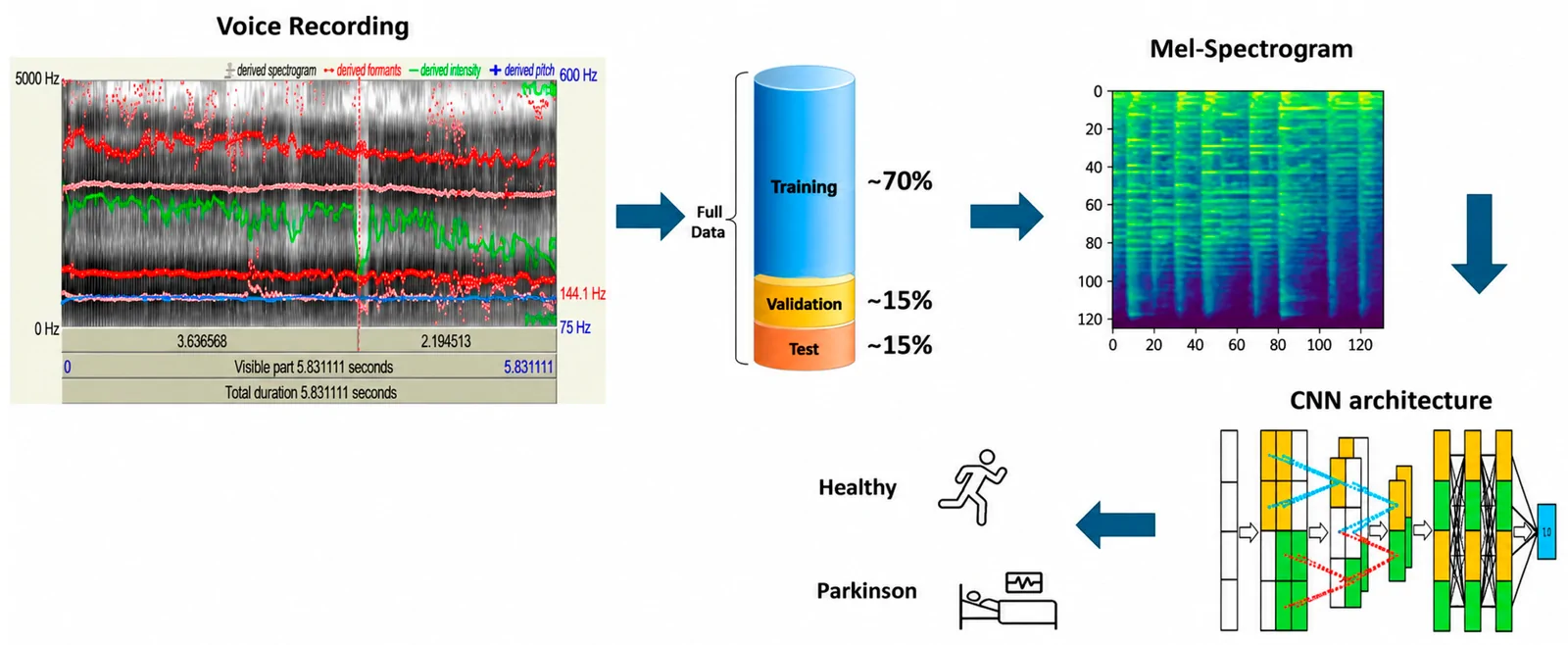
VGGish-CNN-based pipeline for Parkinson’s disease (PD) voice classification, from voice recording acquisition to model performance evaluation.

Hardware and software components were combined to acquire and analyze voice recordings from the PD and CG participants. Controlled voice acquisition was performed at Vito Fazzi Hospital in Lecce, Italy, with a RØDE NT-USB microphone (made in Sydney, Australia), and an IsoVox vocal booth (manufactured in Halmstad, Sweden). Participants were asked to produce a sustained /a/ phonation, which was used as the speech task for the subsequent analyses. As illustrated in Figure 1, the deep-learning workflow was organized into five consecutive stages. In the first stage, the acquired voice signals were organized for further processing. Second, the dataset was randomly divided into subsets to ensure an unbiased evaluation of the model. Third, the audio recordings were transformed into Mel-spectrogram representations, which provide a time-frequency visualization of speech signals and serve as suitable inputs for deep-learning architectures. Fourth, the generated Mel-spectrograms were fed into the pre-trained VGGish convolutional neural network (VGGish-CNN), which automatically extracted high-level acoustic embeddings without the need for manual feature engineering. Finally, the performance of the proposed framework was evaluated using standard classification metrics, including accuracy, precision, recall, F1-score, and the area under the receiver operating characteristic curve (ROC-AUC), to assess its ability to discriminate between PD and CG voice recordings.

### 2.1. RØDE Microphone Description

The RØDE NT-USB is a condenser microphone designed for high-quality digital audio recording. Equipped with an integrated analog-to-digital conversion system and USB interface, the device enables direct connection to computers and compatible mobile platforms without requiring additional recording hardware. Its large-diaphragm condenser design provides high audio sensitivity and clarity, making it well-suited for capturing speech signals with excellent fidelity and minimal distortion [26].

### 2.2. Procedures for Collecting Data

The original voice files were stored in MPEG-4 Audio (M4A) format on the POHEMA website, which is hosted by GPI. The website supports only the M4A format. Therefore, the M4A files were converted to Waveform Audio File Format (WAV) before preprocessing and analysis. The recordings were obtained from Vito Fazzi Hospital in Lecce (Italy), and the study was approved by the Lecce Ethics Committee (Register No.: 17032/2023). The vocal data included 8 PD patients and 8 CG. Each participant produced a sustained /a/ phonation of approximately 6–16 s, acquired at 22,050 Hz and stored using a 32-bit floating-point representation. The recordings collected cross-sectional analysis at the recording level over six months. Overall, 96 voice recordings were obtained from the CG and 93 from the PD group. One participant with PD provided nine rather than twelve recordings because the follow-up period ended after 4.5 months. For participants with PD, the initial recording session was conducted at Vito Fazzi Hospital through the GPI-developed TALIA-WEB platform, using a RØDE microphone and an IsoVox vocal booth to minimize environmental noise. The setup also included the Stand Set configuration, equipped with a height-adjustable speaker stand (Millenium BS2011 MK II; Thomann GmbH, Burgebrach, Germany), after recruitment via the Pohema website (GPI server) where total voice recordings were downloaded from the server. Each participant had a unique identifier, referred to as a tax code (Codice Fiscale in Italian), which enabled the identification and tracking of individual recordings. The remaining recordings were acquired remotely at home twice monthly using the TALIA smartphone application. CG participants followed the same mobile-recording procedure, whereas the initial hospital session was performed only for the PD group. Participant selection for the PD group was performed by a neurologist at Vito Fazzi Hospital, as summarized in Table 1.

### 2.3. Voice Preprocessing

The resultant wav files were preprocessed in two stages: first using Audacity (version 3.7.0), and then Praat (version 6.4.34). In Audacity, background noise was removed, and in Praat, the silent parts of the waveform were trimmed after importing from Audacity. All speech signals were normalized to the range [−1, 1]. Interestingly, 189 final voice samples were obtained after preprocessing with Audacity and Praat: 93 from PD 96 from CG.

The 189 original recordings were randomly assigned to training, validation, and test subsets in five repeated data splits, using proportions of 70%, 15%, and 15%, respectively. Segmentation was carried out only after the recordings had been assigned to the corresponding subsets. Recordings within each subset were then divided independently into non-overlapping 2.5 s segments, producing 777 segments in total. Therefore, all segments originating from a given recording remained within the same subset, preventing that recording’s segments from appearing across training, validation, and test sets; the distribution of participants and segments is illustrated in Table 2. However, because partitioning was performed at the recording level rather than the participant level, different recordings from the same participant could occur in different subsets [26]. The next step for our data was based on preparing raw voice data for use on VGGish-CNN models integrated with XAI. The dataset therefore comprised three hierarchical statistical units: 16 participants (8 PD and 8 CG), 189 original recordings (93 PD and 96 CG), and 777 speech segments. Because repeated recordings from the same participant could occur in different subsets, the resulting segments should not be considered statistically independent at the participant level. The next step involved preparing the raw voice data for use with the VGGish-CNN model integrated with XAI.

### 2.4. Convolutional Neural Network (CNN)

**Figure 2:**
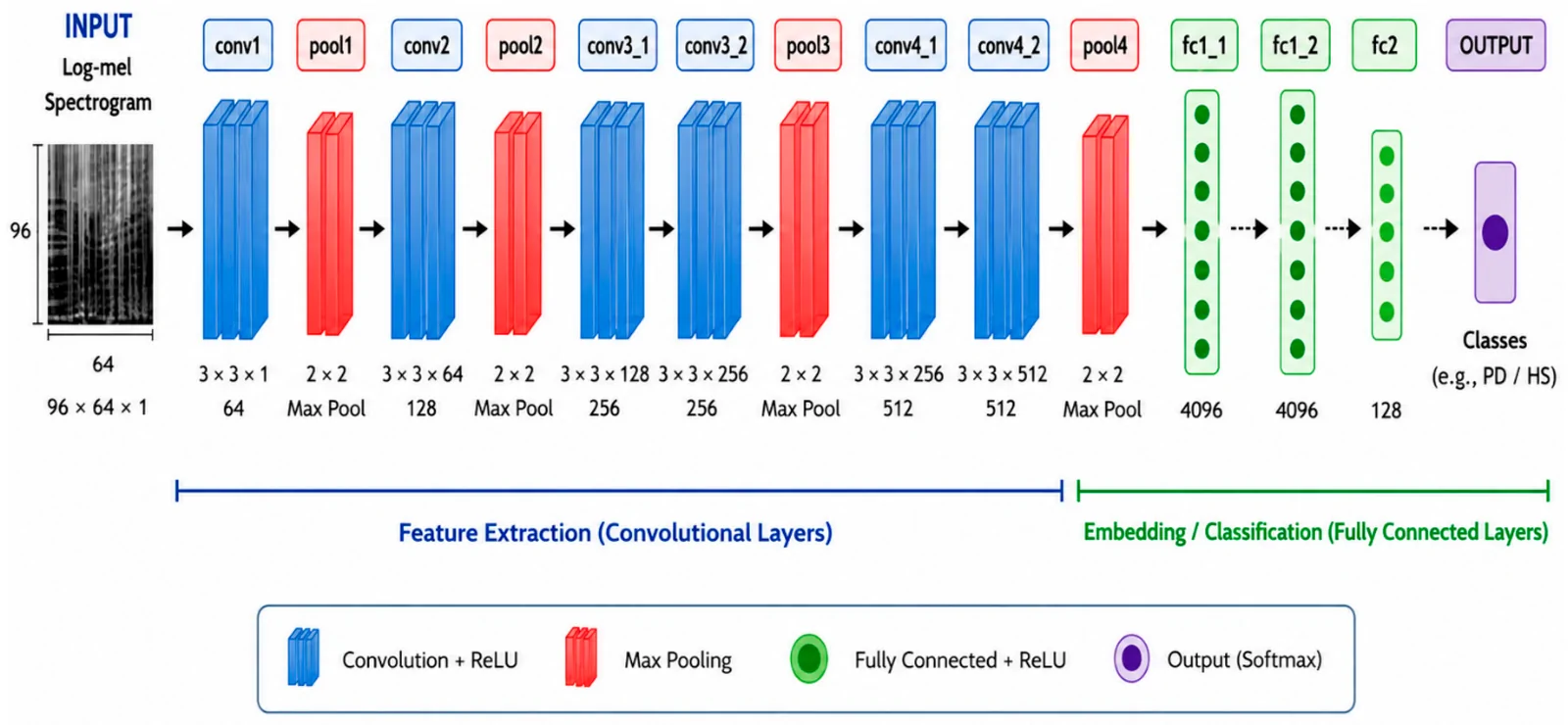
Architecture of the VGGish-CNN model showing convolutional and max-pooling layers for spectro-temporal feature extraction, followed by the custom fully connected layer for PD/CG binary classification.

CNN is a deep-learning framework that learns the task-relevant image representations through a sequence of digital filters, whose parameters are optimized during training. In this research, we used the pre-trained VGGish-CNN architecture on the AudioSet database for the classification of spectrogram images and extraction of features from log-mel spectrogram inputs [27]. This CNN architecture originates from the original VGG network. The AudioSet authors modified it by removing the last group of convolution and max-pooling layers, instead leaving four such groups, as opposed to five. Also, the 1000-unit fully connected (FC) layer in the original visual geometry group (VGG) model was replaced by a 128-unit FC layer, serving as the embedding layer in VGGish according to Figure 2:

The architecture has been selected to exploit transfer learning and make use of its already attained high accuracy and efficiency for specialized voice classification tasks. By doing so, the pre-trained model captures the complex spectro-temporal patterns autonomously, and our pipeline focuses on classifying PD patients from CG. For classification, spectrograms of the sustained vowel /a/ were analyzed from a dataset comprising eight patients with Parkinson’s disease (PD) and eight control group (CG) participants. As mentioned previously, the data were randomly split, and no image augmentation techniques were applied in order to preserve the clinical integrity of the voice biomarkers. The original VGGish network classification head was replaced with a custom multilayer perception consisting of batch normalization, followed by a dense layer with 128 units and a dropout layer of 0.5. This model was trained with the Adam optimizer using a batch size of 32 and a learning rate of 0.0001. Model performance in this paper has been evaluated using five independent train–validation–test splits. The proposed VGGish-CNN demonstrated a mean ROC-AUC of 0.987 for five voice splits, reflecting excellent discriminative capability in the case of acoustic distinctions of PD versus CG samples. Apart from AUC, other performance metrics like accuracy, recall, loss, precision, and the F1-score were also computed.

### 2.5. Comparison with Acoustic-Feature-Based Machine Learning

To provide a baseline context for the proposed VGGish-CNN framework, its performance was descriptively compared with conventional machine-learning models previously evaluated using the same clinical cohort and voice recordings. The machine-learning pipeline extracted handcrafted acoustic representations, including long-term, short-term, and nonlinear features, followed by SHAP-based feature selection and classification using several algorithms, including Random Forest (RF), Support Vector Machine (SVM), K-Nearest Neighbors (KNN), Artificial Neural Network (ANN), and Gradient Boosting (GB). In contrast, the VGGish-CNN framework operates on spectrogram representations and uses pre-trained acoustic representations, thereby avoiding explicit handcrafted acoustic feature extraction and SHAP-based feature selection.

The machine-learning study employed repeated random train–test partitioning, with internal five-fold cross-validation (K = 5) performed within the training set, whereas the present VGGish-CNN framework used repeated independent train–validation–test splits. Given these differences in the split-data and validation procedures, the comparison was intended to provide descriptive context rather than a controlled head-to-head benchmark and should not be interpreted as evidence that either approach is superior to the other.

### 2.6. Explainable Artificial Intelligent: LIME and Occlusion Sensitivity

LIME and Occlusion Sensitivity were applied as XAI methods to facilitate interpretation of the VGGish-CNN predictions. The resulting heatmaps provide visual information about the spectrogram regions that influence individual PD/CG classifications. Both XAI analyses were performed across the five data splits, with LIME and Occlusion Sensitivity heatmaps generated for the evaluated samples [28].

## 3. Results

### 3.1. Study Population

The study initially enrolled 11 participants with PD and 11 CG participants from the University of Salento and Vito Fazzi Hospital in Lecce under the protocol approved by the Lecce Ethics Committee (Register No. 17032/2023). Following participant attrition, the final dataset comprised eight participants with PD and eight CG participants, contributing 93 and 96 voice recordings, respectively, as reported in Table 3.

In our cohort of subjects, which consisted of *n* = 8 PD, and *n* = 8 CG, the Parkinson’s group showed mild to moderate motor impairment (UPDRS = 14 ± 4.83, H&Y = 1.75 ± 0.59). The PD group showed mild-to-moderate motor impairment, with a mean UPDRS score of 14 ± 4.83 and a mean Hoehn and Yahr stage of 1.75 ± 0.59, as summarized in Table 3. All eight participants with PD also presented clinically documented voice alterations. A logistic regression model based exclusively on age and sex was additionally evaluated to examine possible demographic confounding. As reported in Table 4, this model yielded an AUC of 0.844, an accuracy of 0.75, and a balanced accuracy of 0.75. These results show that demographic variables alone contained considerable discriminatory information between the two groups. Therefore, age and sex may have contributed to the overall classification performance observed in the proposed VGGish-CNN model.

### 3.2. VGGish-CNN Classification Results

In our model, feature extraction was carried out automatically. The VGGish-CNN model was applied to our raw voice data, which was preprocessed using Audacity software to remove background noise. The dataset was randomly partitioned into three sets (train, test, val) using a five independent train–validation–test splits strategy to preserve class distribution. This procedure was independently repeated five times to generate five train–validation–test splits, and performance metrics were subsequently summarized across the five test sets. Because data partitioning was performed at the original recording level before segmentation, segments derived from the same original recording were not shared across the training, validation, and test subsets. However, recordings from the same participant could occur in different subsets because participant-level partitioning was not performed. This approach mitigates the risk of overfitting and provides a more reliable estimate of the model’s performance.

**Figure 3:**
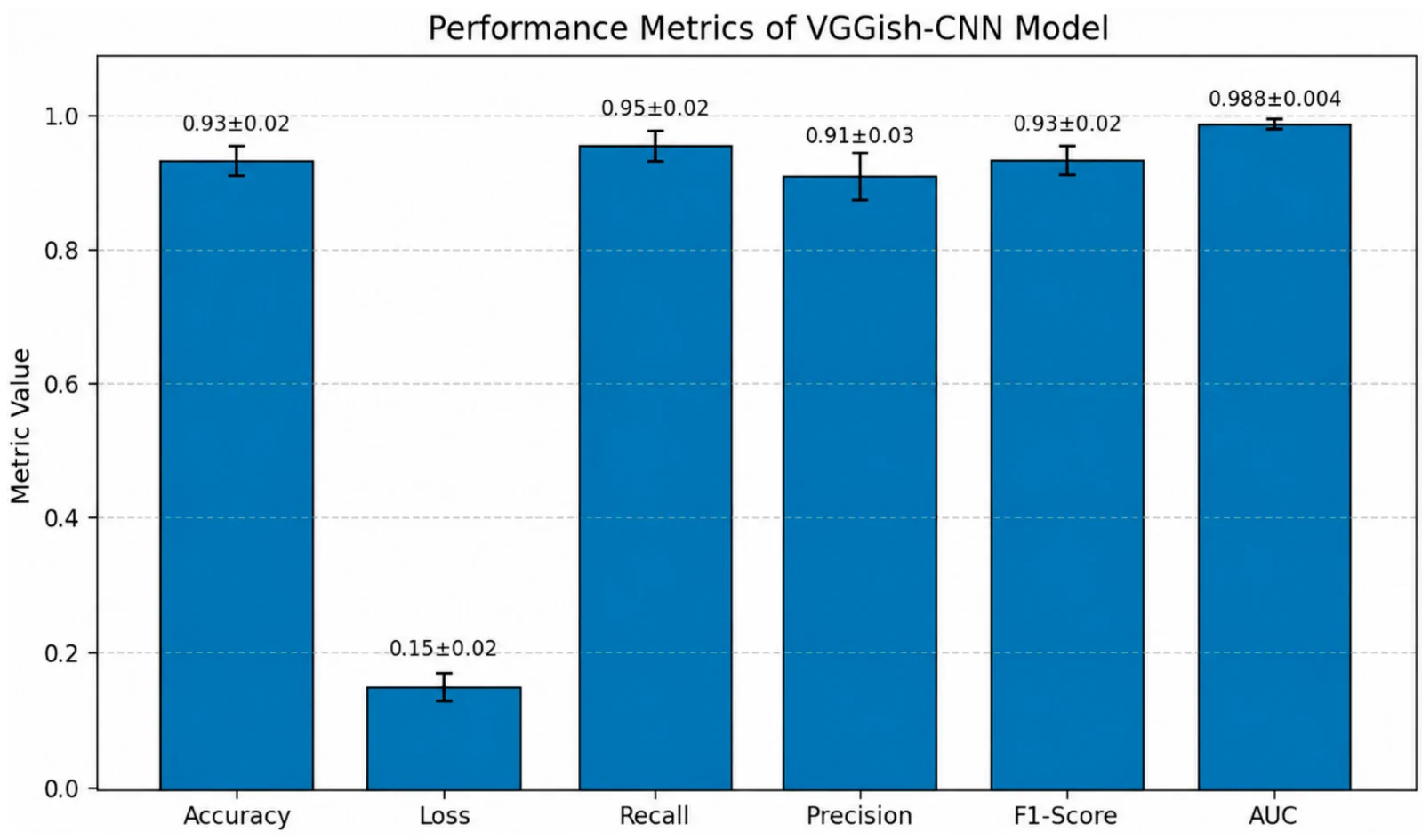
Mean performance metrics of the VGGish-CNN model across five independent train–validation–test splits. Error bars indicate the standard deviation (SD) across the five splits.

In our model, important metrics were assessed using the VGGish-CNN model, which demonstrated the highest classification accuracy for distinguishing Parkinson’s patients from CG. In our study, accuracy, loss, recall, precision, F1-score, and AUC were achieved, as shown in the bar graph presented in Figure 3.

**Figure 4:**
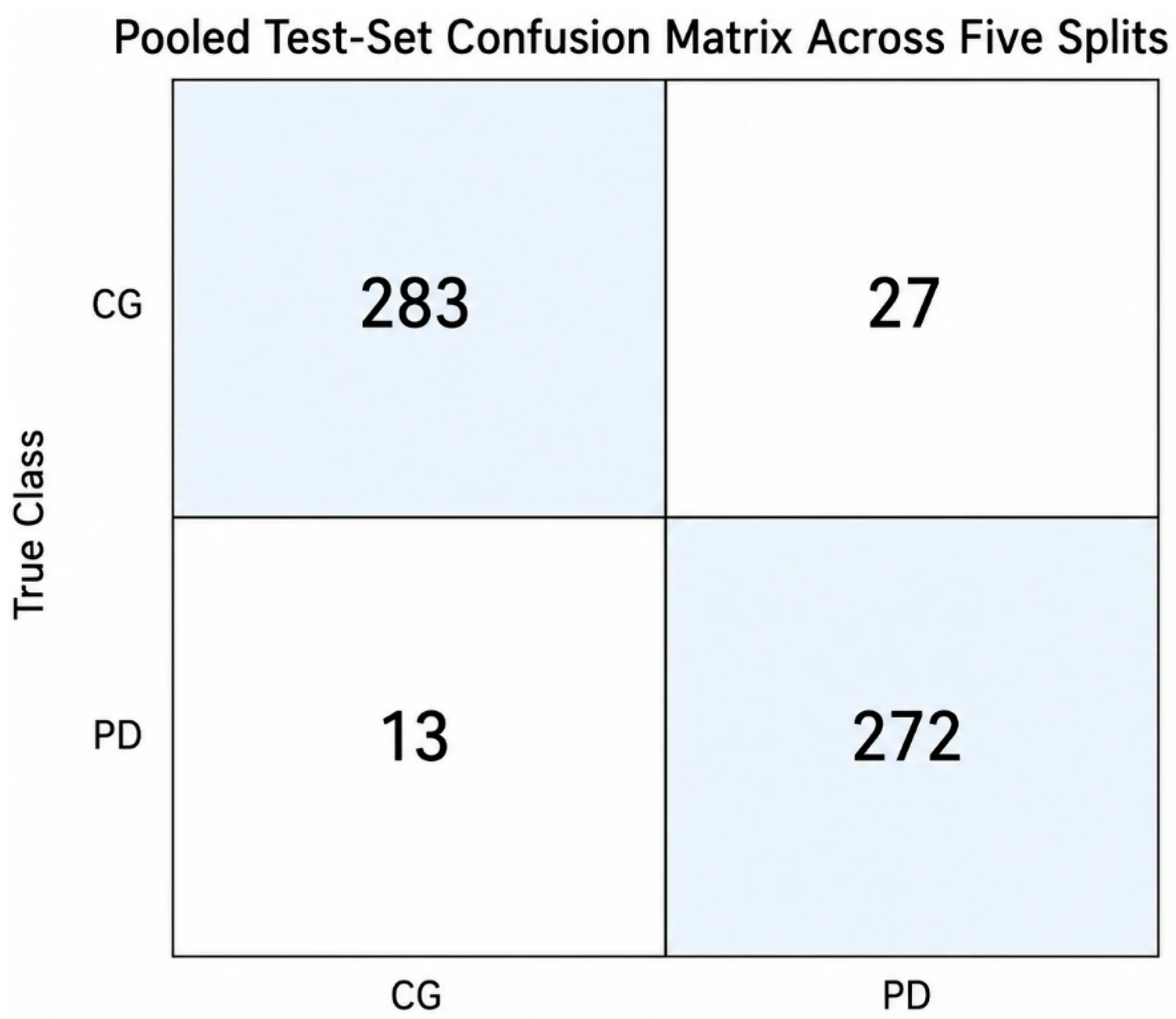
Pooled test-set confusion matrix of the VGGish-CNN model across the five independent train–validation–test splits. The matrix represents the summed classification counts obtained from the test sets across all five splits, with CG and PD indicating the control group and Parkinson’s disease, respectively.

Figure 4 presents the pooled test-set confusion matrix across the five independent train–validation–test splits. Across the five test evaluations, the model correctly classified 283 CG segments and 272 PD segments, while 27 CG segments were misclassified as PD and 13 PD segments were misclassified as CG. These results are consistent with the high overall classification performance of the VGGish-CNN model. Because the confusion matrix pools predictions across five independent random splits, the values represent cumulative test-set classifications across the five evaluations rather than counts of unique recordings or participants.

**Figure 5:**
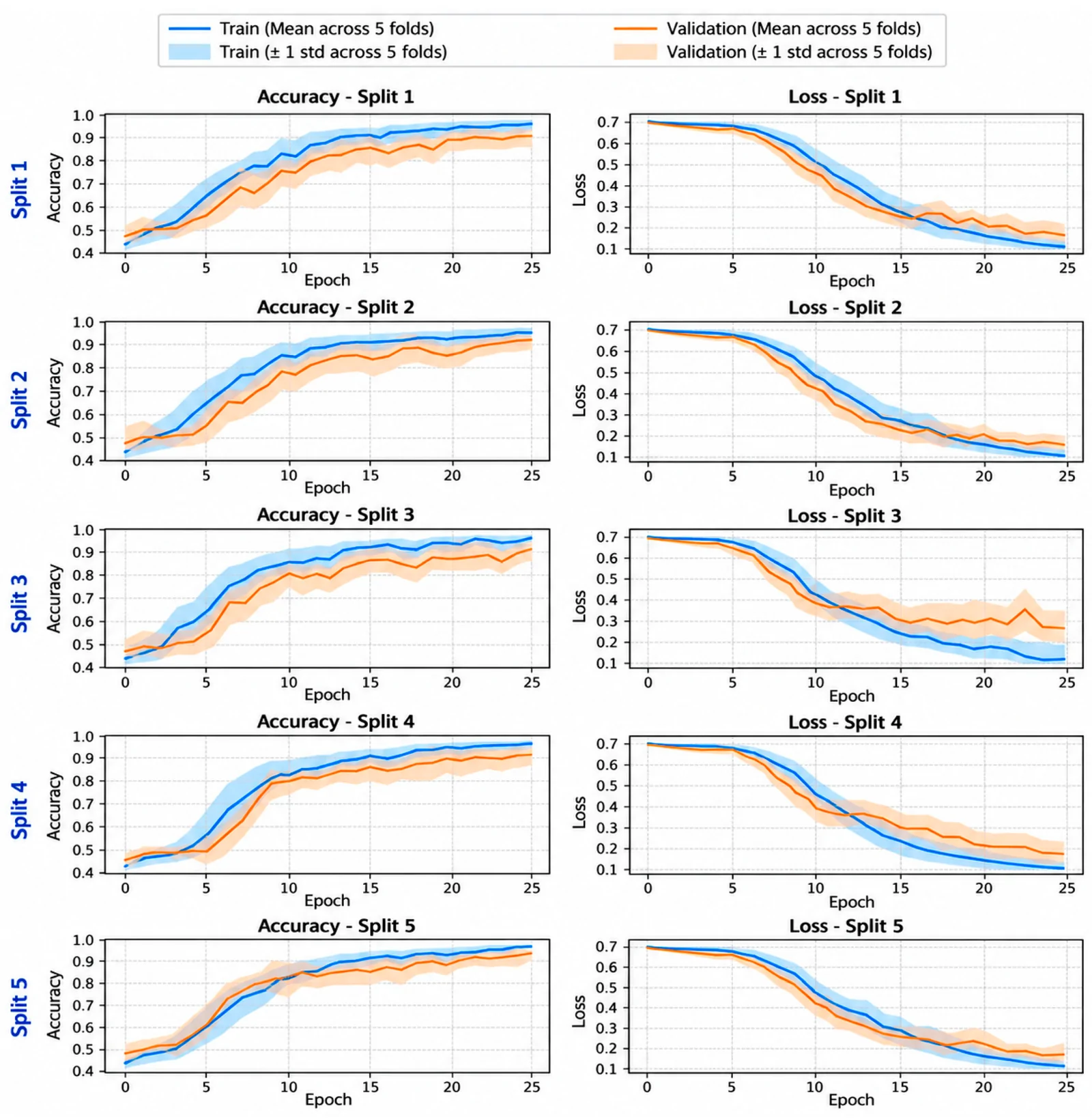
Training and validation performance metrics for splits of the VGGish- CNN.

The proposed deep-learning model achieved an overall accuracy of 0.93, demonstrating excellent classification performance in discriminating between PD and CG. Accuracy, which represents the proportion of correctly classified samples among all evaluated instances, is one of the most widely used performance metrics for assessing classification models. The obtained results suggest that the model captured spectro-temporal patterns that contributed to distinguishing PD and CG voice within the present dataset. However, these patterns should not be interpreted as PD-specific vocal biomarkers without validation in larger independent cohorts. The cross-entropy loss was 0.16, which was relatively low, indicating that the model’s predictions for PD and CG were close to the actual labels; they were shown in Splits 1–5 in Figure 5 which illustrates the training and validation accuracy and loss trajectories across the five cross-validation splits. Across all splits, training accuracy steadily improved over successive epochs, while training loss consistently declined, reflecting stable optimization and effective feature learning.

Validation accuracy exhibited a comparable upward trend, achieving performance levels closely aligned with the training curves in most cases. Although slight fluctuations were observed in some splits particularly Split 2 and Split 3, the overall behavior indicates consistent and stable convergence. Similarly, validation loss generally decreased throughout training, with minor temporary increases that stabilized in later epochs. Notably, there was no pronounced gap between training and validation curves, indicating the absence of substantial overfitting.

The expected inverse relationship between accuracy and cross-entropy loss was clearly demonstrated: as accuracy increases, loss decreases, indicating enhanced predictive confidence and alignment between predicted probabilities and true labels. Overall, these learning curves demonstrated training stability and performance across all five cross-validation splits.

Another important metric for evaluating detection performance is recall, particularly in detecting PD cases correctly. In fact, the recall rate was 0.95, which is a key factor for identifying true positives (Parkinson’s cases) among all actual Parkinson’s patients. The precision, F1-score, and AUC were 0.91, 0.93, and 0.987, respectively.

The model achieved a mean AUC of 0.987 across the five recording-level evaluations, indicating high discrimination between PD and CG samples within the present dataset. However, this result should be interpreted in the context of the small participant cohort and recording-level partitioning and should not be interpreted as participant-level diagnostic performance. This value was slightly higher than those reported in other studies in terms of AUC. Although Iyer et al. did not mention some additional metrics, Shen et al. reported key measures of classification performance. The observed AUC was numerically higher than the values reported in these studies. However, direct comparison should be interpreted cautiously because of differences in datasets, sample sizes, validation strategies, and model architectures.

### 3.3. Contextual Comparison with Acoustic-Feature-Based Machine Learning

Table 5 showed that the acoustic-feature-based machine-learning models and the proposed VGGish-CNN achieved broadly comparable recording-level classification performance. ANN achieved the highest accuracy (0.9545), while KNN achieved the highest ROC-AUC (0.9893). The VGGish-CNN achieved an accuracy of 0.9328 and ROC-AUC of 0.9879. Therefore, these results do not demonstrate superior classification performance of VGGish-CNN over the traditional machine-learning models. Instead, VGGish-CNN achieved competitive performance while using pre-trained spectro-temporal representations without requiring explicit handcrafted acoustic feature extraction and SHAP-based feature selection [26].

### 3.4. Explainable Artificial Intelligence (LIME, Occlusion Sensitivity)

LIME and Occlusion Sensitivity were used as complementary XAI approaches for interpreting individual model predictions. These techniques visualize areas of the input that contribute to a prediction, typically presenting their influence through graphical or heatmap-based explanations [28].

**Figure 6:**
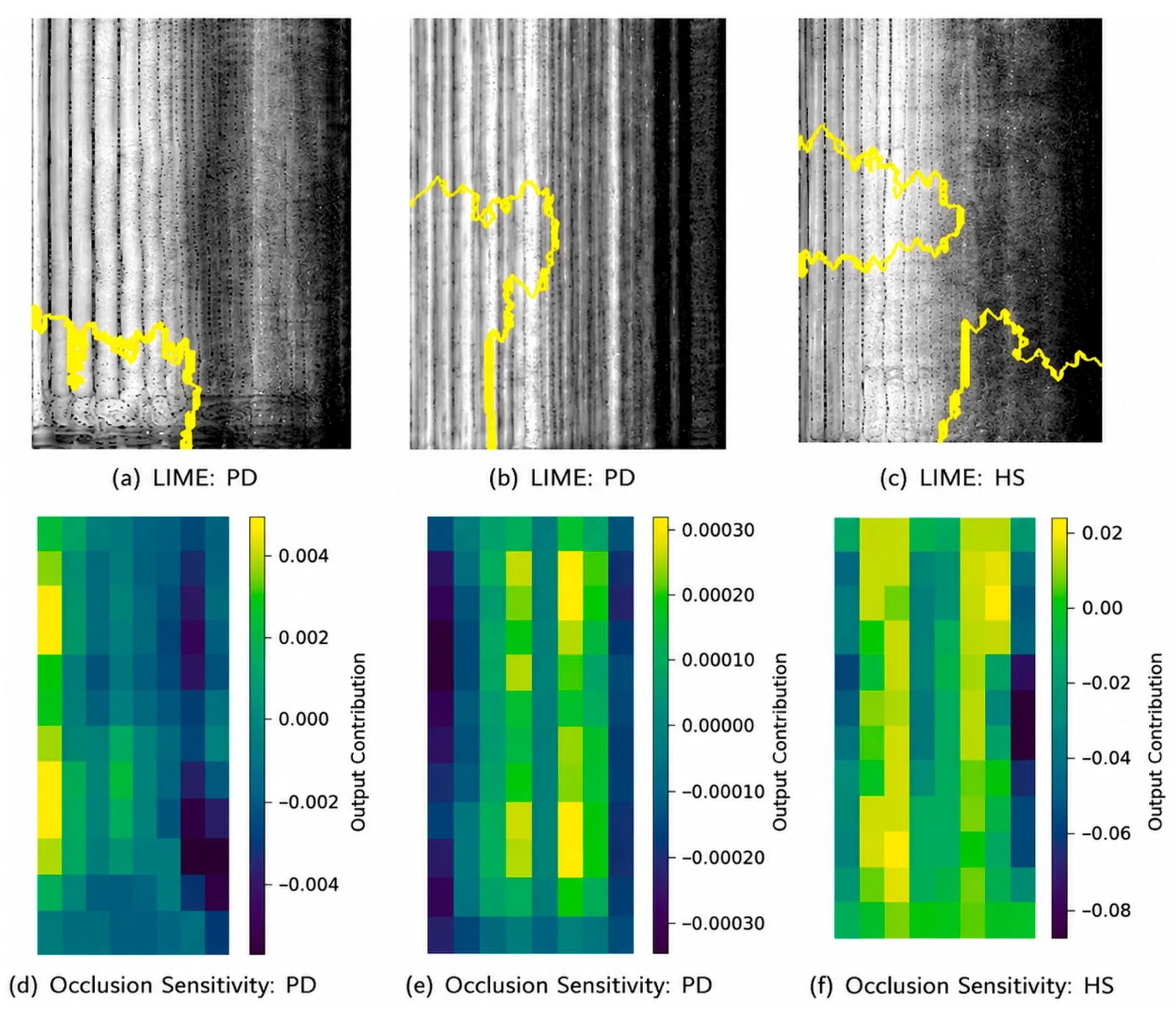
Representative XAI visualizations comparing LIME (**a**–**c**) and Occlusion Sensitivity (**d**–**f**) for voice spectrograms from participants with Parkinson’s disease (PD) and the control group (CG).

To improve the transparency of our predictions, we applied both LIME and Occlusion Sensitivity analysis to identify salient features in the voice samples. Sample heatmaps from these interpretability methods are displayed in Figure 6.

Figure 6 presents representative examples of the XAI analysis obtained using LIME and Occlusion Sensitivity for PD and CG voice spectrograms. In the LIME visualizations (Figure 6a–c), the yellow boundaries identify spectro-temporal regions that contributed to the model prediction for each individual sample. These highlighted regions indicate areas considered locally important by LIME. However, they should not be interpreted directly as specific acoustic or clinical biomarkers. Accordingly, the present XAI analysis should be considered qualitative and exploratory. A systematic participant-level analysis across a larger cohort would be required to evaluate the consistency of these XAI patterns across individuals and to determine whether recurrent spectro-temporal regions correspond to established acoustic markers associated with PD.

In the Occlusion Sensitivity maps (Figure 6d–f), the color bar represents the change in model output produced by systematically occluding different spectro-temporal regions of the input. Regions associated with larger changes in model output indicate areas in which the prediction was more sensitive, whereas regions producing smaller changes had less influence on the prediction. Because the contribution values were calculated independently for each spectrogram, the magnitude and range of the color scale may differ among individual samples.

Overall, these visualizations provide qualitative insight into model behavior. As only representative examples are presented, they cannot establish consistent PD-specific or clinically meaningful biomarkers. A systematic analysis across a larger number of participants and recordings would be required to determine whether recurrent patterns are consistently associated with PD-related voice impairment.

## 4. Discussion

Consistent with previous voice-based studies [14], sustained /a/ recordings were examined using a recording-level cross-sectional design over the six-month collection period. Voice samples from both groups were obtained through the TALIA smartphone and web-based platforms during the collection period. Recordings were sampled at 22,050 Hz, yielding a total of 777 voice segments from eight PD and eight CG participants. Our methodology incorporated two primary components. First, given the limited dataset, we employed the pre-trained VGGish convolutional neural network to enhance feature extraction from raw audio. This model was selected because it was trained by Google on a large and diverse audio corpus, enabling effective transfer learning.

The integration of LIME and Occlusion Sensitivity provided qualitative, sample-specific visualization of spectro-temporal regions influencing the CNN predictions. However, these highlighted regions should not be interpreted as stable PD-specific or clinically validated vocal biomarkers because their consistency across participants was not systematically evaluated in the present pilot study.

Comparison with previously evaluated acoustic-feature-based machine-learning approaches provides further context for interpreting the performance of the proposed framework. The conventional models, i.e., ANN and KNN, showed similar or slightly better results than VGGish-CNN. So, the current results do not support a claim of numerical superiority of deep learning over conventional machine learning. The difference between the two techniques is a significant one in feature representation. The traditional machine-learning pipeline needs to manually extract acoustic features and then use SHAP-based feature selection while VGGish-CNN exploits pre-trained representations learned directly from spectrogram inputs. Therefore, the possible strength of the suggested system is in its ability to cut down reliance on manual feature engineering while still competing at a high level with regard to recording classification accuracy. However, since the two pipelines were based on data-splitting, additional tests need to be carried out using the same partitioned data in order to measure the value of transfer learning.

In contrast, Iyer et al. [11] neither included XAI methods nor used the same transfer-learning strategy. Our VGGish-CNN architecture is also structurally simpler than their Inception-based models, making it more suitable for small to medium datasets.

Our model achieved a higher AUC within the present dataset than the AUC reported in that study. However, because the studies used different datasets and validation procedures, these results should not be interpreted as evidence of model superiority.

Although their grayscale AUC was 0.96, our results achieved 0.98. Their findings were not based on a cross-sectional analysis at the recording level study. Instead, their study was cross-sectional in nature. Our study contributes primarily by integrating VGGish-based transfer learning with XAI techniques for recording-level PD/CG voice classification and by providing qualitative interpretation of individual predictions using LIME and Occlusion Sensitivity.

In some research using machine-learning and deep-learning approaches, internal cross-validation was applied without clearly specifying which samples belonged to the train or test groups, and the datasets were sometimes mixed during implementation. For example, in the study by Iyer et al., they created a dataset consisting of 40 PD and 41 CG, but they did not clearly explain which samples were PD or CG on their dataset when applying their deep-learning or machine-learning methods. In other words, they used the dataset without telling us which individuals belong to the 20% test set, and which belong to the 70% training set [11]. In contrast, in our study, we have split the data into three separate folders: train, test, and validation, and our raw voice samples were clearly labeled to show which recordings belong to Parkinson’s patients with train, test and validation folders, and which belong to participants without PD.

Shen et al. published a paper on explainable artificial intelligence in which they used Iyer’s voice dataset. Apart from the lack of continuous data collection over several months, they also used a combination of models with SHAP, which is typically used for feature engineering, while in our case the feature engineering was performed automatically [29]. Additionally, we used LIME and Occlusion Sensitivity as our XAI methods, and as mentioned before, they did not use transfer-learning methods such as Inception-3.

Eri Kiyoshige et al. [30] conducted a cross-sectional study evaluating prediction models for cognitive impairment based on voice biomarkers. Their approach involved developing both XGBoost-based baseline models, and deep neural network (DNN) models using voice data from 979 participants. Although their framework incorporated advanced machine-learning techniques, they did not apply XAI methods to interpret the internal behavior of the DNN. As a result, the decision-making processes within the hidden layers remain opaque. In contrast, our use of LIME and Occlusion Sensitivity provides qualitative insight into model behavior by highlighting spectro-temporal regions contributing to individual predictions. Furthermore, their reported AUC values were lower than those achieved in our study.

Suppa et al. explained that voice impairment was manifested at an early stage of Parkinson disease within a period of time according to UPDRS, but in their research they used machine-learning models and considered taking L-Dopa, and they figured out that there could be a relationship between improving voice and taking medication, but it could not be totally restored [12]. Although their approach could be considered, our results indicate a new approach in CNN deep learning that could be pre-trained by VGGish models with high accuracy. This study possesses several key strengths. First, voice samples were acquired using the TALIA smartphone and web-based system developed by GPI S.p.A. Second, repeated recordings were obtained through the TALIA platform during the six-month collection period, resulting in multiple samples from each participant. Clinical measures, including UPDRS and Hoehn and Yahr scores, were used to characterize the PD cohort; however, the present analysis did not evaluate longitudinal changes or disease progression. Third, the VGGish-CNN model demonstrated promising recording-level classification performance within this pilot dataset. However, given the small number of participants, recording-level partitioning, and absence of external validation, these findings should be considered preliminary and should not be interpreted as evidence of state-of-the-art performance. Finally, to address the inherent black-box nature of deep-learning models, we incorporated XAI. This crucial integration allowed us to interpret the model’s decisions, enabling the identification of specific Regions of Interest (ROI) in the spectrograms for enhanced prediction of PD relative to CG.

## 5. Conclusions

This pilot study evaluated a VGGish-CNN transfer-learning framework integrated with XAI for recording-level classification of voice samples from participants with PD and CG. The model demonstrated promising classification performance within the present dataset, while LIME and Occlusion Sensitivity provided qualitative insight into the spectro-temporal regions contributing to individual predictions. Although the proposed VGGish-CNN model did not outperform all traditional machine-learning methods, it performed comparably at the level of recordings while being able to automatically learn the acoustic representations. However, given the small number of participants, recording-level partitioning, and absence of external validation, these findings should be considered preliminary and should not be interpreted as evidence of participant-level diagnostic performance. Future studies using larger and more diverse cohorts, strict participant-level validation, and external datasets are required to determine the generalizability and potential clinical utility of this approach for PD screening.

## 6. Limitation

The main limitation of this pilot study is the small number of participants. The final cohort included only eight participants with PD and eight participants in the control group (CG), providing 93 and 96 original voice recordings, respectively. Although the original recordings were split into training, validation, and testing subsets and subsequently segmented into 777 non-overlapping voice segments, the relatively large number of segments does not compensate for the limited number of independent participants. Therefore, the reported model performance should be interpreted cautiously. In addition, the status of the CG was based on self-report rather than neurological examination. Consequently, the absence of PD or other conditions affecting voice could not be clinically confirmed.

A second limitation concerns the generalizability of the results to routine clinical settings. Voice-based assessment is attractive because it is non-invasive, inexpensive, and suitable for remote acquisition. However, similar acoustic abnormalities can occur in neurological, respiratory, laryngeal, age-related, and other conditions. Furthermore, recording conditions in real-world settings may vary substantially with respect to microphones, background noise, recording protocols, medication status, comorbidities, language, and disease severity. The high classification performance observed in the present study may therefore be optimistic when applied to broader and more heterogeneous populations. Accordingly, the proposed framework should currently be regarded as a potential screening or clinical decision-support approach rather than a standalone diagnostic tool. Larger multicenter studies and external validation under routine clinical conditions are required before clinical implementation.

Demographic differences between the groups represent an additional potential source of confounding. Participants with PD were older on average than those in the CG, and the sex distribution was also imbalanced (CG: 6 male/2 female; PD: 4 male/4 female). Because both age and sex can influence voice characteristics, part of the observed classification performance may reflect demographic differences rather than PD-specific vocal alterations. Although age and sex were not used directly as inputs to the deep-learning model, their influence cannot be completely excluded. Sex-stratified analyses were not feasible because of the small cohort size. Future studies should therefore recruit larger, age-matched and sex-balanced populations.

An additional limitation concerns the XAI analysis. LIME and Occlusion Sensitivity were used to provide qualitative, sample-specific explanations, and only representative examples are presented. Therefore, the highlighted spectro-temporal regions cannot be considered stable or PD-specific vocal biomarkers. Future studies with larger participant-level datasets should systematically evaluate the consistency of XAI patterns across participants and investigate whether recurrent regions correspond to established acoustic markers associated with PD.

An additional limitation concerns heterogeneity in recording devices and acquisition environments. The initial PD recordings were acquired at the hospital using a RØDE microphone and IsoVox vocal booth, whereas subsequent PD recordings and control-group recordings were primarily obtained using the mobile-based protocol. Therefore, acquisition-related characteristics may have contributed to the observed classification performance, and the present analysis cannot fully determine whether the learned spectro-temporal patterns reflect disease-related vocal characteristics or differences in recording conditions. Future studies should employ standardized recording devices and environments across groups or perform controlled device-matched analyses to evaluate this potential confounding effect.

Finally, data partitioning was performed at the recording level rather than the participant level. Segmentation was performed only after the original recordings had been assigned to the training, validation, and testing subsets, preventing segments from the same recording from appearing in multiple subsets. Nevertheless, because multiple recordings were available from the same participant, different recordings from one participant could occur in different subsets. Consequently, participant-specific characteristics may have contributed to model performance, and participant-level information leakage cannot be completely excluded. Future studies should employ strict subject-level validation strategies, such as group-based cross-validation or leave-one-subject-out validation, to provide a more rigorous estimate of generalizability to unseen participants.

## Figures and Tables

**Table 1:** Overview of voice recording acquisition, including expected and collected recordings for PD and CG.

Recording Summary	Parkinson Patients (*n* = 8)	Control Group (*n* = 8)
Expected recordings	96	96
Actual recordings collected	93	96

**Table 2:** Distribution of participants and audio segments across the five data splits.

Split	Subset	CG Participants	PD Participants	CG Audio Segments	PD Audio Segments	Total Audio Segments
Split 1	Training	8	8	282	261	543
Split 1	Validation	8	8	60	55	115
Split 1	Test	8	8	62	57	119
Split 2	Training	8	8	282	261	543
Split 2	Validation	8	8	60	55	115
Split 2	Test	8	8	62	57	119
Split 3	Training	8	8	282	261	543
Split 3	Validation	8	8	60	55	115
Split 3	Test	8	8	62	57	119
Split 4	Training	8	8	282	261	543
Split 4	Validation	8	8	60	55	115
Split 4	Test	8	8	62	57	119
Split 5	Training	8	8	282	261	543
Split 5	Validation	8	8	60	55	115
Split 5	Test	8	8	62	57	119

**Table 3:** Characteristics of the study participants.

	CG (*n* = 8)	PD (*n* = 8)
**Demographics:** **Sex (male/female)**	6/2	4/4
**Age at enrolment (years)**	57.5 ± 7.38	72.37 ± 6.44
**Clinical Features (PD group only): Hoehn & Yahr (H&Y) stage of PD**	–	1.75 ± 0.59
**Clinical Features (PD group only): UPDRS**	–	14 ± 4.83
**Disease duration (years)**	–	7.88 ± 4.49
**Voice change (8 patients)**	–	8/8

**Table 4:** Age- and sex-only sensitivity analysis for assessing demographic confounding.

Model	Predictors	Validation	AUC	Accuracy	Balanced Accuracy
Logistic Regression	Age + sex	Leave-one-participant-out	0.844	0.75	0.75

**Table 5:** Comparison of machine-learning approaches and the proposed VGGish-CNN model.

Model	Input	Feature Engineering	Accuracy	ROC-AUC
Random Forest	Acoustic features	SHAP	0.9313	0.9801
KNN	Acoustic features	SHAP	0.9494	0.9893
ANN	Acoustic features	SHAP	0.9545	0.9882
VGGish-CNN	Mel-spectrogram	Pre-trained automatic representation	0.9328	0.9879

## Data Availability

The data supporting the findings of this study can be obtained from the corresponding author upon reasonable request and in accordance with applicable ethical and institutional guidelines. Owing to privacy and confidentiality restrictions associated with clinical voice recordings from human participants, the raw datasets are not publicly accessible. To enhance transparency, reproducibility, and open scientific research, the Python (3.10.12 and 3.12.8) scripts and source code used for data preprocessing, machine-learning model development, and performance assessment have been made freely available through the GitHub repository: https://github.com/mehdi1366-com, accessed on 8 September 2026.
